# Changes in the gut microbiome of patients with type a aortic dissection

**DOI:** 10.3389/fmicb.2023.1092360

**Published:** 2023-02-22

**Authors:** Fei Jiang, Meiling Cai, Yanchun Peng, Sailan Li, Bing Liang, Hong Ni, Yanjuan Lin

**Affiliations:** ^1^Department of Cardiac Surgery, Union Hospital, Fujian Medical University, Fuzhou, China; ^2^Department of Nursing, Union Hospital, Fujian Medical University, Fuzhou, China; ^3^Fujian Provincial Special Reserve Talents Laboratory, Union Hospital, Fujian Medical University, Fuzhou, China; ^4^Department of Physical Examination, Union Hospital, Fujian Medical University, Fuzhou, China

**Keywords:** aortic dissection, gut microbiome, 16S rDNA sequencing, metabolomics, SCFAs

## Abstract

**Objective:**

To investigate the characteristic changes in the gut microbiota of patients with type A aortic dissection (AAD) and provide a theoretical basis for future microbiome-oriented interventional studies.

**Methods:**

High-throughput 16S rDNA sequencing was performed on the stool samples of patients with and without (healthy control subjects) AAD. Using alpha and beta diversity analysis, we compared the gut microbiota composition of 20 patients with AAD and 20 healthy controls matched for gender, age, BMI, and geographical region. The accuracy of AAD prediction by differential microbiome was calculated using the random forest machine learning model. Targeted measurement of the plasma concentration of short-chain fatty acids (SCFAs), which are the main metabolites of the gut microbiome, was performed using gas chromatography–mass spectrometry (GC–MS). Spearman’s correlation analysis was conducted to determine the relationships of gut microbiome and SCFAs with the clinical characteristics of subjects.

**Results:**

The differences in gut microbiota alpha diversity between patients with AAD and the healthy controls were not statistically significant (Shannon index: *p* = 0.19; Chao1: *p* = 0.4); however, the microbiota composition (beta diversity) was significantly different between the two groups (Anosim, *p* = 0.001). *Bacteroidota* was enriched at the phylum level, and the SCFA-producing genera *Prevotella*, *Porphyromonas*, *Lachnospiraceae*, and *Ruminococcus* and inflammation-related genera *Fenollaria* and *Sutterella* were enriched at the genus level in the AAD group compared with those in the control group. The random forest model could predict AAD from gut microbiota composition with an accuracy of 87.5% and the area-under-curve (AUC) of the receiver operating characteristic curve was 0.833. The SCFA content of patients with AAD was higher than that of the control group, with the difference being statistically significant (*p* < 0.05). The different microflora and SCFAs were positively correlated with inflammatory cytokines.

**Conclusion:**

To the best of our knowledge, this is the first demonstration of the presence of significant differences in the gut microbiome of patients with AAD and healthy controls. The differential microbiome exhibited high predictive potential toward AAD and was positively correlated with inflammatory cytokines. Our results will assist in the development of preventive and therapeutic treatment methods for patients with AAD.

## 1. Introduction

Type A aortic dissection (AAD) is a rapidly progressive life-threatening cardiovascular disease. Once it occurs, the afflicted person is highly prone to death from aortic rupture, with the mortality rate being as high as 85% (Mangum and Farber, 2020). In China, both the incidence and age of onset of AAD have shown a steady increase over recent years (Wang et al., 2014; Lu et al., 2017), thereby posing a severe threat to public health. However, effective pharmacological treatment methods for AAD are lacking. Surgery remains the only method for the radical treatment of AAD, but it is difficult and has a high risk of death (Bossone et al., 2018). Therefore, new targeting mechanisms are urgently required to provide novel approaches for the prevention and treatment of AAD.

The pathophysiological mechanisms of AAD mainly involve inflammatory cytokines aggregation, extracellular matrix (ECM) degradation, elastic fiber fracture, and vascular smooth muscle cell apoptosis, with inflammation regarded as the key factor promoting AAD progression (Cui et al., 2021; Liu et al., 2022). A study reported that inflammation may induce aortic wall degradation, and the degraded aortic wall fragments may in turn cause the aggregation of proinflammatory cells and production of proteases, which inflicts further injuries to the aortic wall (Pan et al., 2022). It is therefore evident that the inhibition of inflammation may serve as an effective strategy for preventing AAD onset and progression.

A considerable number of studies have demonstrated the roles of gut microbiota in inflammatory cardiovascular diseases. These include direct participation in hypertension (Avery et al., 2022), atherosclerosis (Chen et al., 2021), and post-myocardial infarction inflammatory response (Manolis et al., 2022). Gut microbes mainly affect the host physiological functions through the synthesis of biologically active metabolites, with the potential mechanisms involving inflammation regulation, immune regulation, and oxidative stress (Tang et al., 2019). It is well-known that many types of gut microbiota metabolites, including short-chain fatty acids (SCFAs), trimethylamine-N-oxide, and bile acids, play essential roles in the pathophysiological changes in cardiovascular diseases (Brown and Hazen, 2018). In particular, SCFAs can enter the bloodstream and affect the development, differentiation, and functions of various immune and inflammatory cells (Adnan et al., 2017), as well as participate in the immune and inflammatory responses of the body. Gut microbiota dysbiosis may be involved in the pathological processes of cardiovascular diseases through the disruption of SCFA-producing bacteria and increase in pro-inflammatory bacteria and opportunistic pathogens (Witkowski et al., 2020).

Research on the potential roles of the gut microbiome in AAD pathogenesis is lacking. The changes and involvement of the gut microbiome in AAD remain unclear, and the determination of gut microbiome characteristics of patients with AAD will help elucidate the role of gut microbiota dysbiosis in AAD pathogenesis. Therefore, we conducted a case–controlled study on 20 patients with AAD and 20 healthy controls. Using 16S rDNA sequencing of their stool samples and targeted metabolomics analysis of their plasma samples, we evaluated the accuracy of differential gut microbiome composition in predicting AAD, and we investigated the relationships of gut microbiota and metabolomic changes with clinical characteristics. The results of this study can provide a scientific basis and novel approach for the prevention and treatment of AAD.

## 2. Methods

### 2.1. Protocol approvals and patient consent

This clinical study was approved by the Ethics Review Committee of Union Hospital Fujian Medical University, Fujian, China (Ethics Approval No. 2022KY083) and registered in the China Clinical Trials Registration Center (No. ChiCTR2200059936). The protocol in this study conformed to the Declaration of Helsinki. Informed consent was obtained from all subjects.

### 2.2. Protocol recruitment of the study subjects

A case–control study was performed at Fujian Heart Medical Center from January 2022 to May 2022. A total of 20 AAD patients (AAD group) were recruited in this study. Twenty healthy controls (control group) matched for gender, age, body mass index (BMI), and geographical region were recruited at the physical examination center during the same period. The inclusion criteria were as follows: (1) age ≥ 18 years; (2) AAD confirmed by computed tomography angiography, magnetic resonance angiography, or ultrasound. The exclusion criteria were as follows: (1) usage of antibiotics, glucocorticoids, immunosuppressants, or probiotics within 3 months before stool sampling; (2) suffering from digestive system tumors, digestive tract infections, inflammatory bowel disease, or other diseases; (3) history of intestinal surgery; (4) history of recurrent diarrhea or constipation within 1 month.

### 2.3. Data collection

The study designed a questionnaire to collect the demographic and clinical information of participants, including gender, age, Body mass index (BMI) and history of smoking and drinking. Systolic blood pressure (SBP), diastolic blood pressure (DBP), white blood cells (WBCs), neutrophils, monocytes, lymphocytes, C-reactive protein (C-rp), glycosylated hemoglobin (HbA1C), total cholesterol (TC), total triglyceride (TG), high-density lipoprotein (HDL), and low-density lipoprotein (LDL) were obtained from medical records.

### 2.4. Fecal DNA extraction and 16S rDNA sequencing

Fecal samples of AAD patients are collected *via* anal swabs by trained nurses after emergency surgical anesthesia, and the healthy people in the physical examination center of our hospital provide fecal samples voluntarily as healthy controls. The specimens were transported to the laboratory in dry ice within 2 h and stored at −80°C. The fecal samples, packed with dry ice, were sent for analyses to the laboratory at LC-Bio Technology Co., Ltd., Hang Zhou, Zhejiang Province, China.

The total genomic DNA was isolated from subjects feces samples using the QIAGEN QIAamp Fast DNA Stool Mini Kit, according to manufacturer’s instructions. Te primer pairs used: 338 forward primer (5′-ACTCCTACGGGAGGCAGCAG-3′) and 806 reverse primer (5′-GGACTACHVGGG TWTCTAAT-3′). The 5′ ends of the primers were tagged with specific barcodes per sample and sequencing universal primers. Polymerase chain reaction (PCR) amplification was performed in a total volume of 25 μL reaction mixture containing 25 ng of template DNA, 12.5 μL PCR Premix, 2.5 μL of each primer, and PCR-grade water to adjust the volume. The V3-V4 hypervariable region of the bacterial 16S rDNA gene was amplified using PCR. The PCR products were confirmed with 2% agarose gel electrophoresis. The PCR products were purified by AMPure XT beads (Beckman Coulter Genomics, Danvers, MA, United States) and quantified by Qubit (Invitrogen, United States). The amplicon pools were prepared for sequencing and the size and quantity of the amplicon library were assessed on Agilent 2,100 Bioanalyzer (Agilent, United States) and with the Library Quantification Kit for Illumina (Kapa Biosciences, Woburn, MA, United States), respectively. The libraries were sequenced on NovaSeq PE250 platform. Analyses of the 16S rDNA microbiome sequencing data was performed using the free online platform of LC-Bio Cloud Platform.^1^ Amplicon sequence variants (ASVs) were used to construct operational taxonomic units (OTUs). The final ASV feature list and feature sequences were obtained, and further diversity analysis, species classification annotation, and difference analysis were conducted. Alpha diversity was evaluated by the species indices (Shannon, Chao 1), and Beta diversity was used to explore the diversity of the microbial structure. Principal coordinate analysis (PCoA) and Analysis of similarities (Anosim) was performed to determine the similarity among the microbial communities in various samples using Vegan v2.5–3 package. The linear discriminant analysis (LDA) effect size (LEfSe) and similarity percentage (SIMPER) were used to identify the differences in taxa between patients with AAD and healthy controls.

### 2.5. Targeted metabolomics for quantitative assessment of plasma

On the day of enrollment, approximately 5 ml of fasting peripheral blood was collected by nurses with unified training with the consent of the subjects. After collection, the blood samples were temporarily stored in a refrigerator at 4°C and then centrifuged within 2 h (9,000 rpm, 4°C, 15 min). The upper layer (plasma) was collected and frozen at −80°C. The blood samples, packed with dry ice, were sent for analyses to the laboratory at LC-BioTechnology Co., Ltd. Then, 100 μL of serum was mixed with 50 μL of the internal standard solution (40 μg/l of acetic acid-d_4_) in a 1.5 ml tube, and 400 μL of methanol was added to the sample. After vortex mixing, the sample was submitted to ultrasound in ice water for 10 min. Then the sample was placed at −20°C for 30 min. After centrifugation (12,000 rpm, 4°C, 5 min), 200 μL of supernatant was obtained. The supernatant was dried with nitrogen, diluted with 200 μL methanol, and subjected to GC–MS analysis according to the manufacturer’s recommendations. The internal standard method was used for quantification, and the retention time and SIM fragment ions were compared by standard methodology. All mass spectrometry data acquisition and quantitative analysis of target compounds were performed using the Thermo Scientific Xcalibur software. The final concentration of the sample was the calculated concentration measured by the instrument multiplied by the dilution factor.

### 2.6. Statistical analysis

SPSS26.0 and R (v.3.5.2) were used for data processing and analysis. Qualitative parameters are expressed as numbers and percentage N (%). The chi-squared test or Fisher exact test was used to test differences between groups. The normally distributed quantitative data are presented as mean ± standard deviation (mean ± SD), and non-normally distributed data are presented as medians and quartiles. Differences between groups were testing using the T-test, Wilcoxon rank sum test, Mann–Whitney U test, and Kruskal–Wallis test. Random forest models were trained with the R random forest package to predict AAD. The correlations between the variables (clinical parameters, differential intestinal microflora, and plasma metabolites) were calculated using Spearman rank correlation analysis. *p* < 0.05 indicated significance. The q-value was calculated to evaluate the false discovery rate (FDR) for correction of multiple comparisons.

## 3. Results

### 3.1. Clinical characteristics

There was no significant difference in age, BMI, gender, and systolic blood pressure between the AAD and control groups (*p* > 0.05). There were also no significant differences in smoking and drinking habits between the two groups. However, the levels of white blood cells (WBCs), C-rp, monocytes, neutrophils, and other related inflammatory factors in AAD group were significantly higher than those in the control group (*p* < 0.05; Table 1). The raw data are presented in Supplementary Table S1.

**Table 1:** Demographic and clinical characteristics of subjects.

Characteristic	Control group *N* = 20	AAD group *N* = 20	F/Z/χ^2^	*p-*value
Age, years	57.85 ± 12.09	60.05 ± 9.91	−0.629	0.533
Gender, n (%)	/	/	0.00	0.999
Male, n (%)	18 (90.0)	18 (90.0)	/	/
Female, n (%)	2 (10.0)	2 (10.0)	/	/
BMI, kg/m^2^	23.15 ± 1.98	24.52 ± 3.66	−1.465	0.154
Smoking, n (%)	11 (55.0)	13 (65.0)	0.417	0.519
Drinking, n (%)	10 (50.0)	9 (45.0)	0.100	0.752
SBP, mm Hg	130.20 ± 12.09	131.65 ± 29.08	−0.206	0.839
DBP, mm Hg	78.45 ± 8.95	75.10 ± 10.84	1.066	0.293
WBCs,10^9/L	5.31 (4.59,6.03)	12.02 (8.64,14.48)	6.006	<0.0001
Neutrophils,10^9/L	2.89 (2.59,3.44)	10.56 (7.90,12.47)	−7.599	<0.0001
Monocytes,10^9/L	0.31 (0.24,0.31)	0.54 (0.34,0.73)	−3.568	0.001
Lymphocytes,10^9/L	1.97 (1.62,1.97)	0.75 (0.55,1.16)	6.336	<0.0001
C-rp, mg/L	0.41 (0.26,1.24)	4.79 (1.88,22.76)	−2.457	<0.0001
HbA1C (%)	5.96 ± 0.83	5.64 ± 0.61	1.370	0.179
TC, mmol/L	5.06 ± 0.91	4.52 ± 0.79	1.996	0.053
TG, mmol/L	1.86 ± 1.08	1.50 ± 0.92	1.121	0.269
HDL, mmol/L	1.26 ± 0.31	1.21 ± 0.30	0.555	0.582
LDL, mmol/L	3.36 ± 0.98	2.52 ± 1.25	2.335	0.025

Continuous and categorical variables are expressed as mean (standard deviation) or median (quartile range) and percentage, respectively. The classified data were tested using the chi-squared test. If they were in line with normal distribution and homogeneity of variance, an independent sample *t*-test was used for the comparison of quantitative data between groups. If the variance is not normal, the rank-sum test was performed. *p* < 0.05 (two-tailed) indicated significance. BMI, body mass index; SBP, systolic blood pressure; DBP, diastolic blood pressure; WBCs, white blood cells; C-rp, C-reactive protein; HbA1C, glycosylated hemoglobin; TC, total cholesterol; TG, total triglyceride; HDL, high-density lipoprotein; LDL, low-density lipoprotein.

### 3.2. Comparison of gut microbiota diversity between type A aortic dissection patients and healthy controls

**Figure 1:**
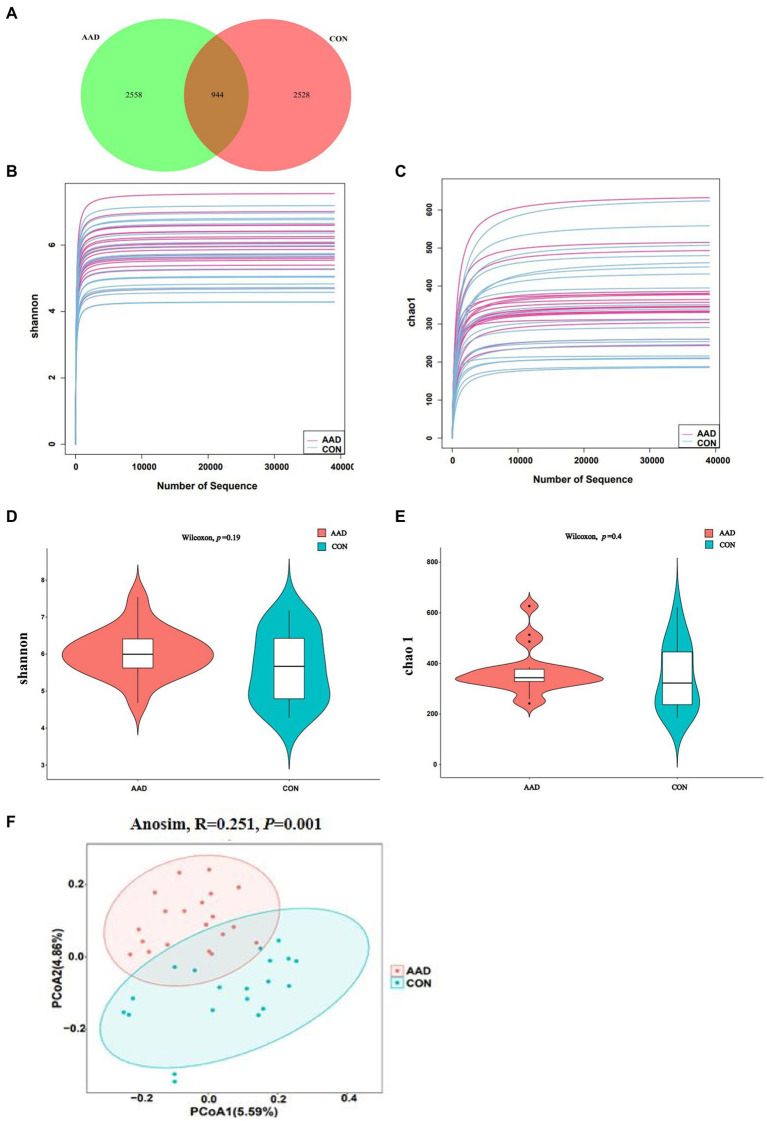
Diversity of the gut microbiota in patients with AAD and healthy controls. **(A)** Venn diagram showing the number of unique and common ASVs between the two groups. **(B,C)** Rarefaction curves of Shannon index and Chao 1. **(D,E)** Comparisons of Alpha diversity as determined by Shannon index and Chao 1 between the AAD and control groups. **(F)** Comparisons of Beta diversity as determined by Jaccard distance between the AAD and control groups. ASVs, amplicon sequence variants; PCoA, principal coordinate analysis; Anosim, analysis of similarities, *N* = 20.

To determine the gut microbiota composition of the 20 patients with AAD and 20 healthy controls, high-throughput 16S rDNA sequencing was performed on all bacteria in the stool samples of the subjects. Using the ASV approach, operational taxonomic unit (OTU) analogues were constructed, and an ASV feature table and characteristic sequences were obtained (Supplementary Table S2). The gut microbiota of the AAD and control groups consisted of 5,086 ASVs in total, with 944 ASVs shared between both groups, 2,558 specific ASVs found only in patients with AAD, and 2,528 ASVs found only in the healthy controls (Figure 1A). Differences in gut microbiota were observed between the two groups, which satisfied the requirements for cluster analysis. Based on the lowest number of sequences, 39,055 sequences were extracted from all samples for alpha diversity analysis. The following parameters were set to produce the dilution curve: --p-max-depth 39,055 --p-min-depth 1 --p-steps 10 --p-iterations 10. The dilution curves of both the Shannon and Chao1 indices exhibited a saturation plateau, with coverage values within the range of 0.9975–0.9998. This indicated that sequencing depth was sufficient to capture the microbial diversity (Shannon index) and richness (Chao1) of the samples (Figures 1B,C). Quantification of the microbial alpha diversity of each subject showed that the microbial diversity and richness were not significantly different between the two groups (*p* > 0.05) (Figures 1D,E). The PCoA based on Jaccard distance matrix revealed that the gut microbiota composition of patients with AAD was different from that of the healthy controls (Beta diversity, Anosim, R = 0.251, *p* = 0.001, Figure 1F).

### 3.3. Taxonomic analysis of the gut microbiota composition of type A aortic dissection patients and healthy controls

**Figure 2:**
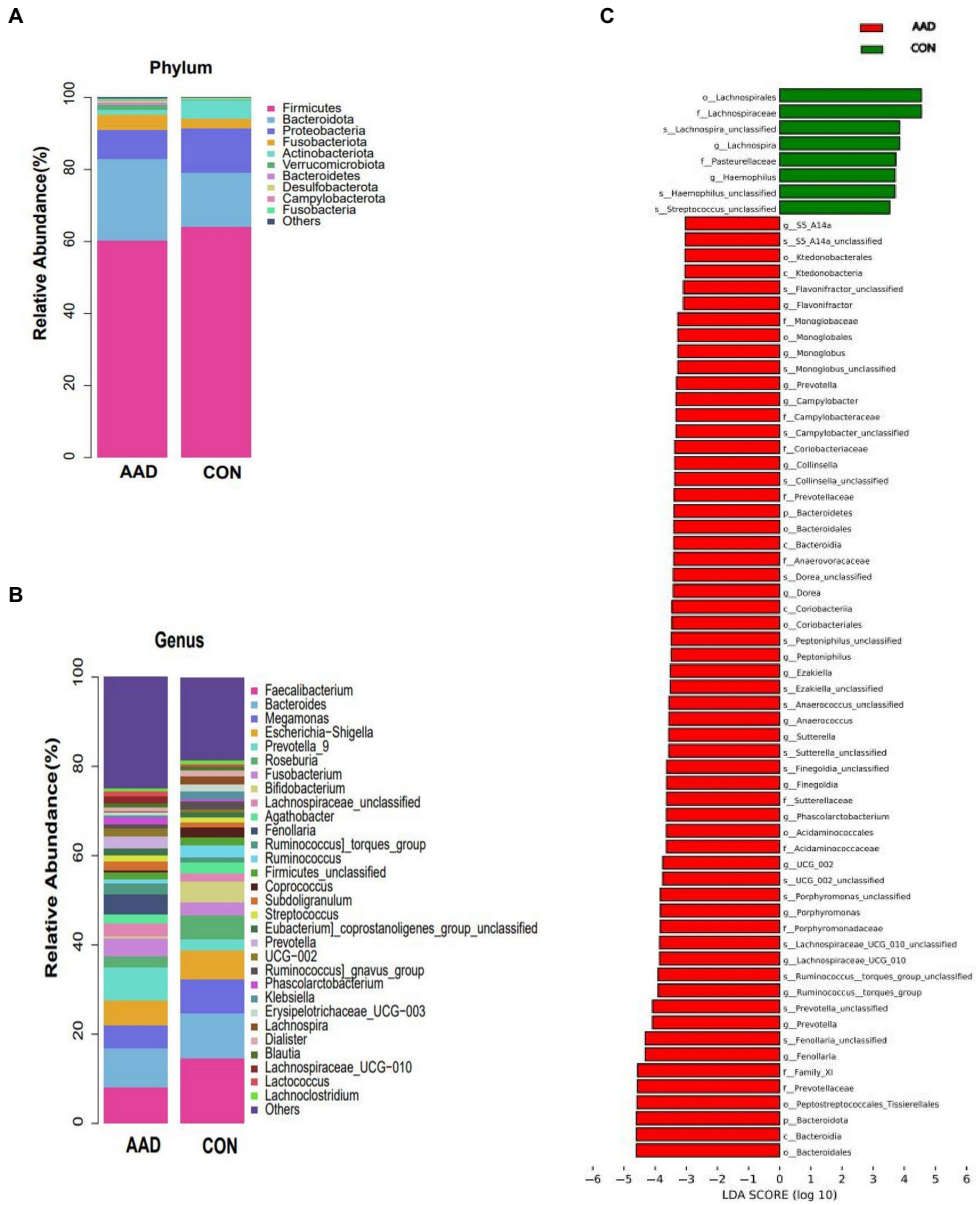
Taxa differences between patients with AAD and healthy controls. Relative abundance of the major bacteria at the **(A)** phylum and **(B)** genus levels. **(C)** Differences in intestinal bacteria between the AAD and control groups. LefSe (LDA score > 3) was used to detect major differences in bacterial taxa between patients with AAD and healthy controls. Red bars are genera with higher relative abundance in patients with AAD. Green bars are genera with higher relative abundance in healthy controls. *X*-axis shows the log LDA scores; LDA, linear discriminant analysis; *N* = 20.

At the phylum level (Figure 2A), *Firmicutes* (average relative abundance: 62.3%) were the most abundant bacteria, followed by *Bacteroidota* (18.8%), *Proteobacteria* (10.3%), *Fusobacteriota* (3.4%), and *Actinobacteriota* (3.2%). The control group had a higher *Firmicutes/Bacteroidota* (F/B) ratio (4.31) than that of the AAD group (2.67). At the genus level (Figure 2B), *Faecalibacterium* was the most common bacterial genus, followed by *Bacteroides*, with the former accounting for a considerably higher percentage of gut microbes in the control group (14.7%) than that in the AAD group (8.9%). *Prevotella* (*p <* 0.0001, FDR corrected *q* = 0.0107), *Porphyromonas* (*p* < 0.0001, FDR corrected *q* = 0.0267), *Fenollaria* (*p* = 0.0007, FDR corrected *q* = 0.0340),*Anaerococcus* (*p* = 0.0005, FDR corrected *q* = 0.0267), *Campylobacter* (*p* = 0.0001, FDR corrected *q* = 0.0107), *Murdochiella* (*p* = 0.0002, FDR corrected *q* = 0.0178) were frequently distributed in AAD gut compared to normal controls (Figure 2C). The presence of bacterial genera with significant differences between the two groups was confirmed using the linear discriminant analysis Effect Size (LEfSe) tool. The results indicated that 8 bacterial taxa were enriched in the control group and 59 were enriched in the AAD group. For example, patients with AAD had higher abundances of *Prevotella* (*p <* 0.0001), *Porphyromonas* (*p* < 0.0001), *Lachnospiraceae* (*p* = 0.0221), *Ruminococcus* (*p* = 0.0265), *Anaerococcus* (*p* = 0.0005), *Fenollaria* (*p* = 0.0007), *Sutterella* (*p* = 0.011), and *Peptostreptococcus* (*p* = 0.0004), and *Haemophilus* (*p* = 0.0126) and *Lachnospiraceae*_NK4A136 (*p* = 0.0172) were higher in the healthy control group (Figure 2C). Supplementary Table S3 shows the bacterial genera were ranked by the contribution degree to distinguish between patients with AAD and healthy controls by using SIMPER analysis, and some of which were also screened by using LEfSe analysis, such as *Ruminococcus*, *Prevotella*, *Porphyromonas*, *Lachnospiraceae*, *Sutterella,* and *Fenollaria*.

### 3.4. Prediction of type A aortic dissection by gut microbiota using the random forest model

**Figure 3:**
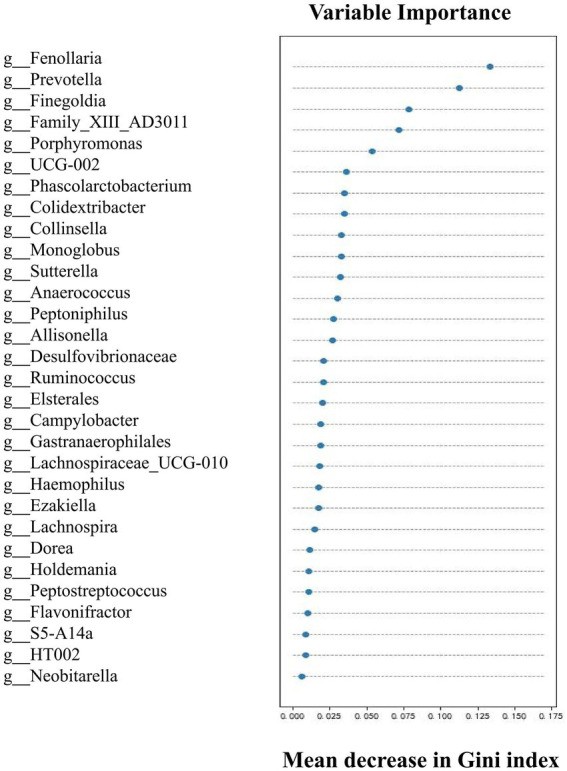
Top 30 genera with great importance in distinguishing patients with AAD from healthy controls by random forest modeling. The 30 most important predictors of AAD vs. healthy were ranked by the Gini index determined from the random forest algorithm trained to distinguish the two cohorts. The taxa are ranked from top to bottom by decreasing Gini index. Since Gini index scores quantify the strength of each respective predictor, the best predictors of AAD are at the top of the plot.

Upon determining the differential taxa of gut microbiota in the two groups, we attempted to ascertain the bacterial groups most closely associated with AAD. The random forest algorithm was used to determine which bacteria had pivotal roles in distinguishing subjects with AAD from the control subjects. Our model contained 186 individual decision trees, ensembled by class designation. Thirty bacterial species with the highest Gini index predicted the presence or absence of AAD in subjects with 87.5% (Figure 3). The AUC of the receiver operating characteristic curve was 0.833 using the random forest model in combination with leave-one-out cross validation. Figure 3 shows the top 30 genera with the greatest importance, some of which were also screened using LEfSe and SIMPER analysis, such as *Fenollaria*, *Prevotella*, *Porphyromonas*, *Lachnospiraceae*, *Sutterella*, and *Ruminococcus*.

### 3.5. Targeted metabolomics reveals elevated plasma short-chain fatty acids concentrations with type A aortic dissection patients

**Figure 4:**
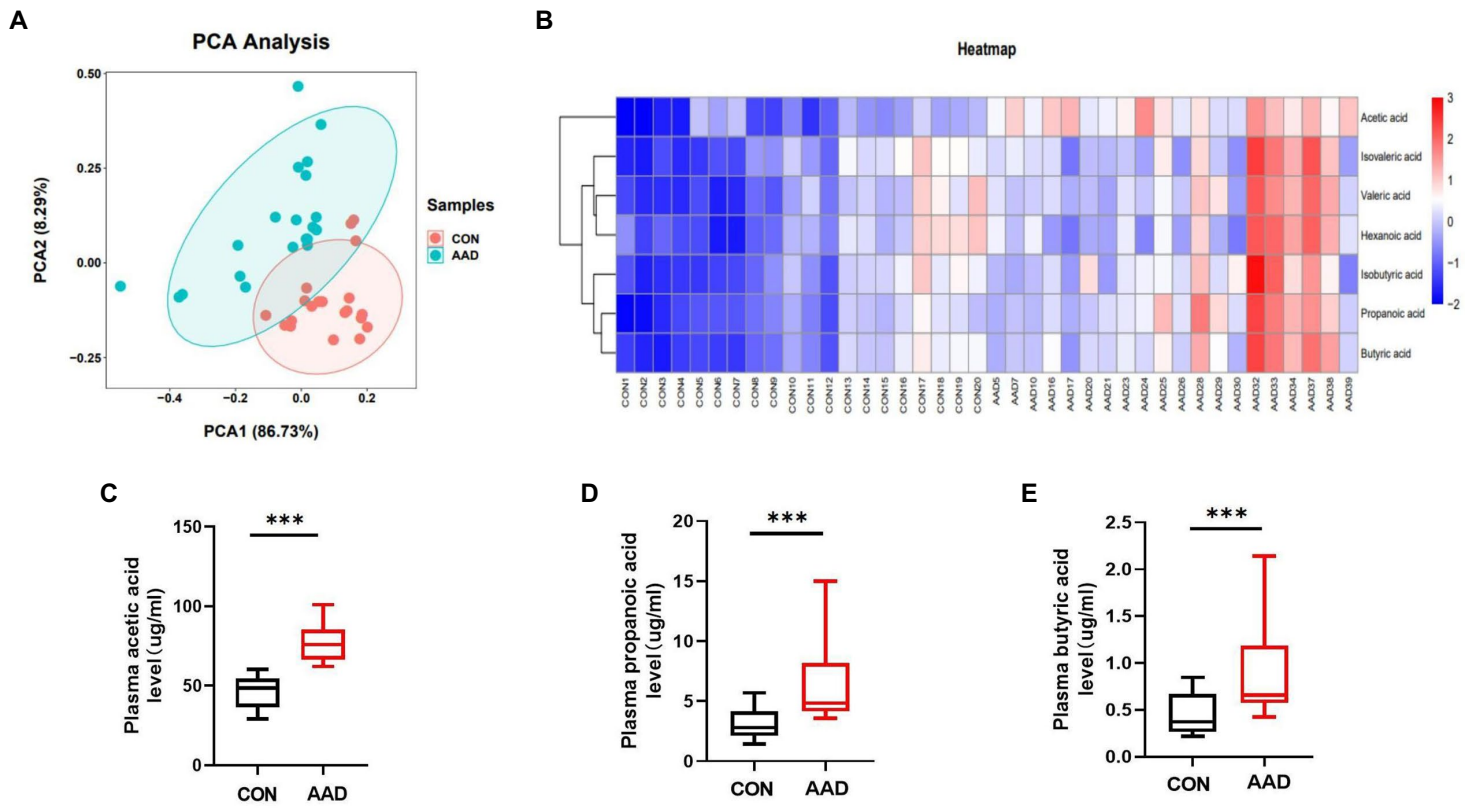
Comparison of plasma SCFAs between patients with AAD and healthy controls. **(A)** Plasma principal component analysis. **(B)** Heatmap clustering. **(C)** Box plots show plasma acetic acid. **(D)** Propanoic acid. **(E)** Butyric acid interquartile spacing and median between the two groups. The comparisons between groups by Wilcoxon rank sum tests; significance is represented as ***p < 0.001, *N* = 20.

The results obtained from dimensionality reduction of data by principal component analysis (PCA) revealed significant differences between samples of the two groups (Figure 4A). Quantitative information of SCFAs was subjected to cluster heatmap analysis, and SCFA expression values in the samples were represented by different shades of color (Figure 4B). The samples were subjected to GC–MS-based metabolomics analysis for the measurement of plasma SCFA content in the two groups. Acetic acid (*p* < 0.0001, Figure 4C), propanoic acid (*p* = 0.0002, Figure 4D), butyric acid (*p* = 0.001, Figure 4E), isobutyric acid (*p =* 0.004, Supplementary Figure S1A), isovaleric acid (*p* = 0.014, Supplementary Figure S1B), valeric acid (*p* = 0.0015, Supplementary Figure S1C), and hexanoic acid (*p* = 0.023, Supplementary Figure S1D). The results indicated that the content of seven types of SCFAs in the plasma was significantly higher in patients with AAD than in the healthy controls.

### 3.6. Relationships of gut microbiome and short-chain fatty acids with clinical characteristics

**Figure 5:**
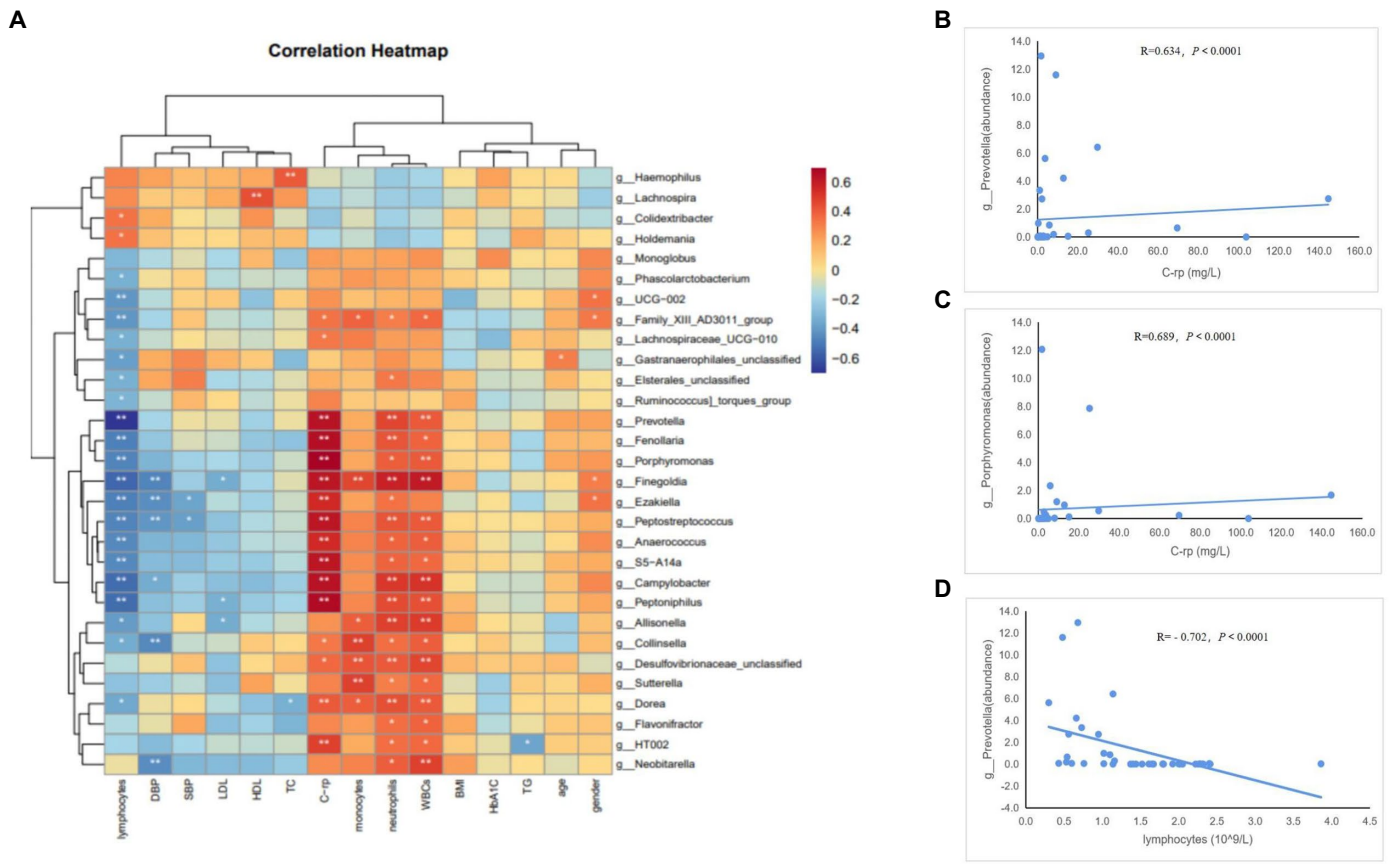
Spearman correlation analysis of different bacteria and clinical characteristics. **(A)** Heatmap showing the correlation between different bacteria and clinical characteristics. **(B)** Scatter plots showing the correlation between C-rp and *Prevotella*. **(C)** Scatter plots showing the correlation between C-rp and *Porphyromonas*. **(D)** Scatter plots showing the correlation between lymphocytes and *Prevotella*. The color intensity represents magnitude of correlation. Red reflects positive correlations, blue reflects negative correlations; *p < 0.05; **p < 0.01.

**Figure 6:**
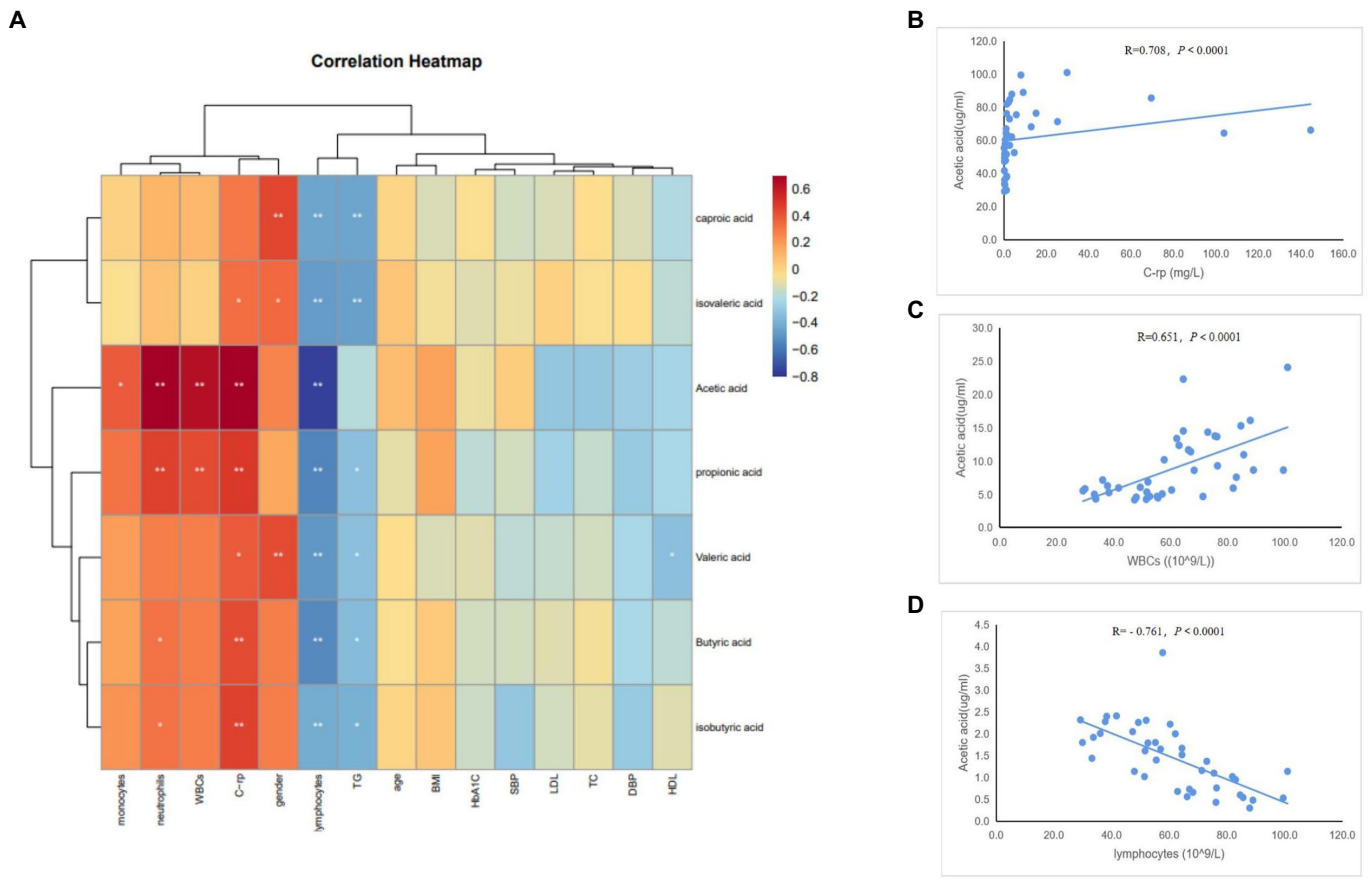
Spearman correlation analysis of different SCFAs and clinical characteristics. **(A)** Heatmap showing the correlation between different SCFAs and clinical characteristics. **(B)** Scatter plots showing the correlation between acetic acid and C-rp. **(C)** Scatter plots showing the correlation between acetic acid and WBCs. **(D)** Scatter plots showing the correlation between acetic acid and lymphocytes. The color intensity represents magnitude of correlation. Red reflects positive correlations, blue reflects negative correlations; *p < 0.05; **p < 0.01.

To determine whether the gut microbiome and its metabolites (SCFAs) were correlated with the clinical characteristics of the subjects, we conducted Spearman’s correlation analysis of the top 30 differential bacterial genera of the gut microbiota and relevant clinical characteristics of the subjects. Results of the heatmap analysis (Figure 5A) showed that the bacterial genera with relatively higher abundances in the AAD group than in the control group were positively correlated with inflammatory markers (C-rp, WBCs, monocytes, and neutrophils) and negatively correlated with lymphocytes (immune factors). The scatter plot of bacterial genera with higher abundances in the gut microbiome (Figures 5B–D) showed that C-rp was positively correlated with the abundances of *Prevotella* (R = 0.634, *p* < 0.0001) and *Porphyromonas* (R = 0.689, *p* < 0.0001). Furthermore, lymphocytes were negatively correlated with the abundance of *Prevotella* (R = -0.702, *p* < 0.0001). Results of the heatmap analysis (Figure 6) revealed that SCFAs were also positively correlated with inflammatory markers and negatively correlated with lymphocytes. Subsequently, a scatter plot of acetic acid, which was the metabolite with the highest content in the gut, was generated (Figures 6B–D). Acetic acid concentration was positively correlated with C-rp (R = 0.708, *p* < 0.0001) and WBCs (R = 0.651, *p* < 0.0001) and negatively correlated with lymphocytes (R = -0.761, *p* < 0.0001).

## 4. Discussion

In this study, differences in the gut microbiome between patients with AAD and healthy controls were confirmed, for the first time, by comparing gut microbiota characteristics of the two groups of subjects. Microbiota uniformity and richness were highly similar between the groups, while beta diversity analysis revealed the presence of significant differences in microbiota composition. The occurrence of AAD is closely associated with high blood pressure, which is evidenced by the fact that more than 80% of patients with AAD have a history of hypertension (Dong et al., 2019). However, the gut microbiome diversity of these patients has not yet been investigated. Therefore, our findings on the gut microbiota diversity were compared with the results of previous hypertension-related studies. We found that the gut microbiomes of AAD patients were similar to those of hypertension patients, while the alpha diversity of gut microbiota in both groups of patients showed a lack of significant differences compared to that of the healthy controls (Calderón-Pérez et al., 2020). This may be ascribed to the facts that gut microbiota is closely associated with lifestyle (Marques et al., 2018) and that both hypertension and AAD are lifestyle-related diseases, which may be the reason for the considerable overlap of microbiota pathogenic factors. However, our results also indicated that beta diversity was significantly different between the AAD and control groups, which was inconsistent with the findings of Calderón-Pérez et al. (2020). This inconsistency may be related to the significant difference in BMI between the hypertensive and control groups of the latter study. Given that BMI may affect gut microbiota composition (Pinart et al., 2021), the subjects of the two groups in this study were matched for BMI, sex, age, and geographical region to reduce the effects of confounding bias. Our results suggest that even though the overall diversity of the gut microbiota was maintained, certain changes had occurred in the stability of gut microbial community structures of patients with AAD.

At the phylum level, *Firmicutes* was the most abundant bacterial group, followed by *Bacteroidota*, *Proteobacteria*, and *Fusobacteriota*. This is in accordance with findings of Yu et al. (2018); however, these authors focused mainly on the differences in gut microbiota structural characteristics of patients with AAD pre-and post-surgery without comparing the gut microbiomes of patients with AAD with those of the healthy control subjects. Our results indicated that the F/B ratio of patients with AAD was significantly lower than that of the healthy controls, which was consistent with the findings of Liu et al. (2021). The F/B ratio is generally regarded as a marker of gut microbiota dysbiosis. Previous research has shown that *Firmicutes* are beneficial bacteria, with intestinal transport promoted by *Firmicutes* genera and inhibited by *Bacteroidota* genera (Parthasarathy et al., 2016). We concluded that the decrease in F/B ratio in patients with AAD may have been caused by the depletion of beneficial bacteria and enrichment of harmful bacteria, which demonstrates that the balance in the gut microbiota was disrupted in patients with AAD.

Using the LEfSe tool, we found that *Bacteroidota* was enriched in the AAD group at the phylum level, and the abundances of the genera *Prevotella*, *Porphyromonas*, *Lachnospiraceae*, *Ruminococcus*, *Fenollaria*, and *Sutterella* were significantly higher in patients with AAD than in the healthy controls. *Bacteroidota* are important symbiotic bacteria in the human colon. In addition to having a supporting role for the host and other microbes in the microecosystem, they profoundly impact host sensitivity to inflammatory and infectious diseases (Sun et al., 2019). Type A aortic dissection is a destructive disease with an extremely high fatality rate. Although its exact pathogenesis has not yet been elucidated, it is known that inflammatory response is the main molecular mechanism that promotes AAD progression (Wang et al., 2021). This is in accordance with our results showing that the level of inflammatory markers was significantly higher in the AAD group than in the control group. This may explain the enrichment of *Bacteroidota* and inflammation-related bacterial genera in patients with AAD.

Interestingly, we found that *Prevotella* was enriched in the patients with AAD in this study. This is contradictory to the findings of other studies on the gut microbiota of populations with cardiovascular diseases, which have generally indicated a decreased abundance of *Prevotella* in the group with the disease compared to that in the control group (Yin et al., 2015; Emoto et al., 2017). This inconsistency may be due to the concurrent presence of glucose metabolism disorders in patients with AAD, as the enhancement of glycolysis may promote disease progression (Xia et al., 2022). Pedersen et al. (2016) reported that *Prevotella copri* upregulates the biosynthesis of branched-chain amino acids in the intestine and subsequently promotes host insulin resistance. The enhanced insulin resistance in turn promotes the occurrence of AAD through the induction of phenotypic switch in vascular smooth muscle cells (Zheng et al., 2022), which may explain the increase in the abundance of *Prevotella* in patient with AAD observed in this study. We calculated the Gini index values for bacterial genera with significant differences between the two groups and adopted the random forest machine learning algorithm to calculate the accuracy of AAD prediction by differential microbiome. Our results indicated that the top 30 bacterial genera with the highest Gini index values predicted the presence or absence of AAD with an accuracy of 87.5%. The AUC of the receiver operating characteristic curve was 0.833. Therefore, it is evident that the unique microbiome characteristics of patients with AAD possess high predictive ability toward AAD. The discovery of this previously unknown role of gut microbiota in AAD may provide a novel perspective for the exploration of methods for the prevention, treatment, diagnosis, and management of AAD.

In recent years, increasing evidence has shown that the gut microbiota is capable of regulating host inflammatory responses through the generation of active metabolites in the intestine. Among the various types of metabolites, SCFAs have attracted interest from researchers. They are the most abundant anions in the colon (Yang et al., 2020) and are absorbed into the human circulatory system. They may participate in inflammation by serving as hormone signals on target tissues and cells (Lymperopoulos et al., 2022). To further investigate the relationship between SCFAs and AAD occurrence and progression, we performed a targeted metabolomics analysis of plasma samples of patients with AAD and healthy controls. Our results indicated that the concentration of SCFAs were higher in the AAD group than in the control group, which was consistent with another finding of this study showing that SCFAs-producing bacterial genera were enriched in patients with AAD (Lau et al., 2017; Santisteban et al., 2017). Previous studies have demonstrated that SCFAs promote the migration of neutrophils toward inflammation sites through an increase in L-selectin expression on neutrophils and CINC-2αβ release, which contributes to inflammatory process initiation (Vinolo et al., 2009). Mills et al. (2006) also observed an increase in SCFAs concentration under inflammatory conditions. However, Yang et al. (2022) presented another perspective, stating that SCFAs exerted protective effects in mouse models of Ca_3_ (PO4)_2-and_ elastase-induced acute abdominal aortic aneurysms and highlighting the significant anti-inflammatory effects of SCFAs in aortic inflammation. It is therefore evident that SCFAs have conflicting roles in inflammation-mediated diseases, which may be manifested as pro-or anti-inflammation. The role of SCFAs in AAD is not sufficiently studied, but the findings of the present study and experimental results of our previous study collectively suggest that the gut microbiota and its SCFAs exert pro-inflammatory effects in AAD.

To validate our conjecture, we performed a Pearson’s correlation analysis to elucidate the relationships between the gut microbiome and SCFAs with clinical characteristics of the patients. Our analysis results revealed that bacteria enriched in AAD and SCFAs were positively correlated with inflammatory markers to varying degrees, demonstrating the relationship between microbiota dysbiosis and inflammation. Interestingly, the bacteria enriched in AAD and SCFAs were also negatively correlated with the abundance of lymphocytes. Given that lymphocyte numbers provide an indirect indication of immune status, such a phenomenon may be related to immune dysfunction caused by the disruption of gut microbiota (Pang et al., 2022).

From the results described above, we deduced that abnormal changes in the gut microbiota of patients with AAD cause metabolism disorders in the gut microbiota, and the inflammatory response elicited by gut microbiota dysbiosis plays a significant role and may be the key mechanism in the occurrence and progression of AAD. However, reports on the roles, molecular mechanisms, and SCFAs produced by gut microbiota in patients with AAD are lacking. Animal and cell experiments are required for further understand this topic, and will serve as the next step in our research.

## 5. Limitations

This study has several limitations: (1) The small sample size of the study may limit its general applicability and generalizability. Therefore, multicenter studies with a large sample size are required for further research; (2) Almost all the patients with AAD, included in the present study, received different antihypertensive medications, and it is currently unclear whether these medications may affect the composition and metabolism of the gut microbiota. Therefore, further exploration into the relevant effects is required; (3) Our discussion was mainly focused on the structure and composition of the gut microbiota without in-depth investigation at the transcriptomic and proteomic levels. This shortcoming should also be addressed in future research.

## 6. Conclusion

In the present study, the presence of unique microbiome characteristics in patients with AAD was demonstrated for the first time. The differential microbiome exhibited extremely high predictive potential toward AAD, with both the microbiome and its metabolites being positively correlated with inflammatory cytokines. Our results highlighted a previously unknown role of the gut microbiome in AAD, which provides a theoretical basis for future microbiome-oriented interventional studies and paves the way for the development of preventive treatment methods for patients with AAD.

## Data availability statement

The original contributions presented in the study are included in the article/Supplementary material, further inquiries can be directed to the corresponding author. The data presented in the study are deposited in the NCBI repository, accession number PRJNA910484.

## Ethics statement

The investigation conformed with the principles outlined in the Declaration of Helsinki and was approved by the Hospital Research Ethics Committee (Ethics Approval No. 2022KY083). Informed consent was obtained from patients before this study.

## Author contributions

YL and FJ conceived the whole study. FJ drafted the manuscript and analyzed the data. MC, YP, SL, BL, and HN were in charge of data collecting. All authors provided an important revision of this manuscript.

## Funding

This research was funded by the Fujian Provincial Finance Special Project (grant number 2021XH019) and China Nursing Association 2021 Annual Research Project (grant number ZHKY202110).

## Conflict of interest

The authors declare that the research was conducted in the absence of any commercial or financial relationships that could be construed as a potential conflict of interest.

## Publisher’s note

All claims expressed in this article are solely those of the authors and do not necessarily represent those of their affiliated organizations, or those of the publisher, the editors and the reviewers. Any product that may be evaluated in this article, or claim that may be made by its manufacturer, is not guaranteed or endorsed by the publisher.

## Footnotes

^1^ cloud.LC-Bio.com
