# Supplementary figures and images for: Changes in the gut microbiome of patients with type a aortic dissection
Source: Front Microbiol. 2023 Feb 22;14:1092360. doi: 10.3389/fmicb.2023.1092360 (PMC9992204; doi:10.3389/fmicb.2023.1092360)

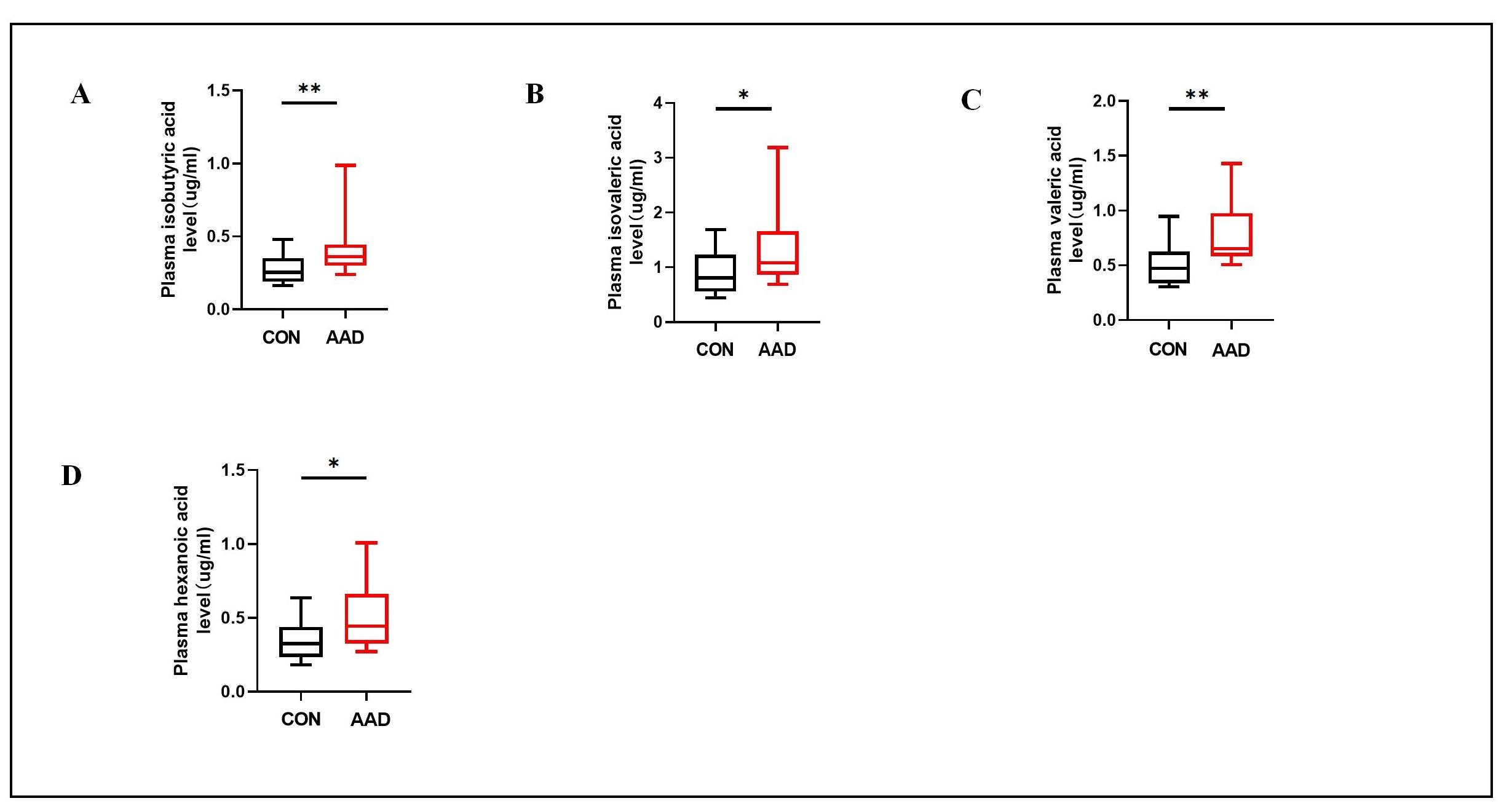

Supplement: Supplementary file 4 [file Image_1.jpg]

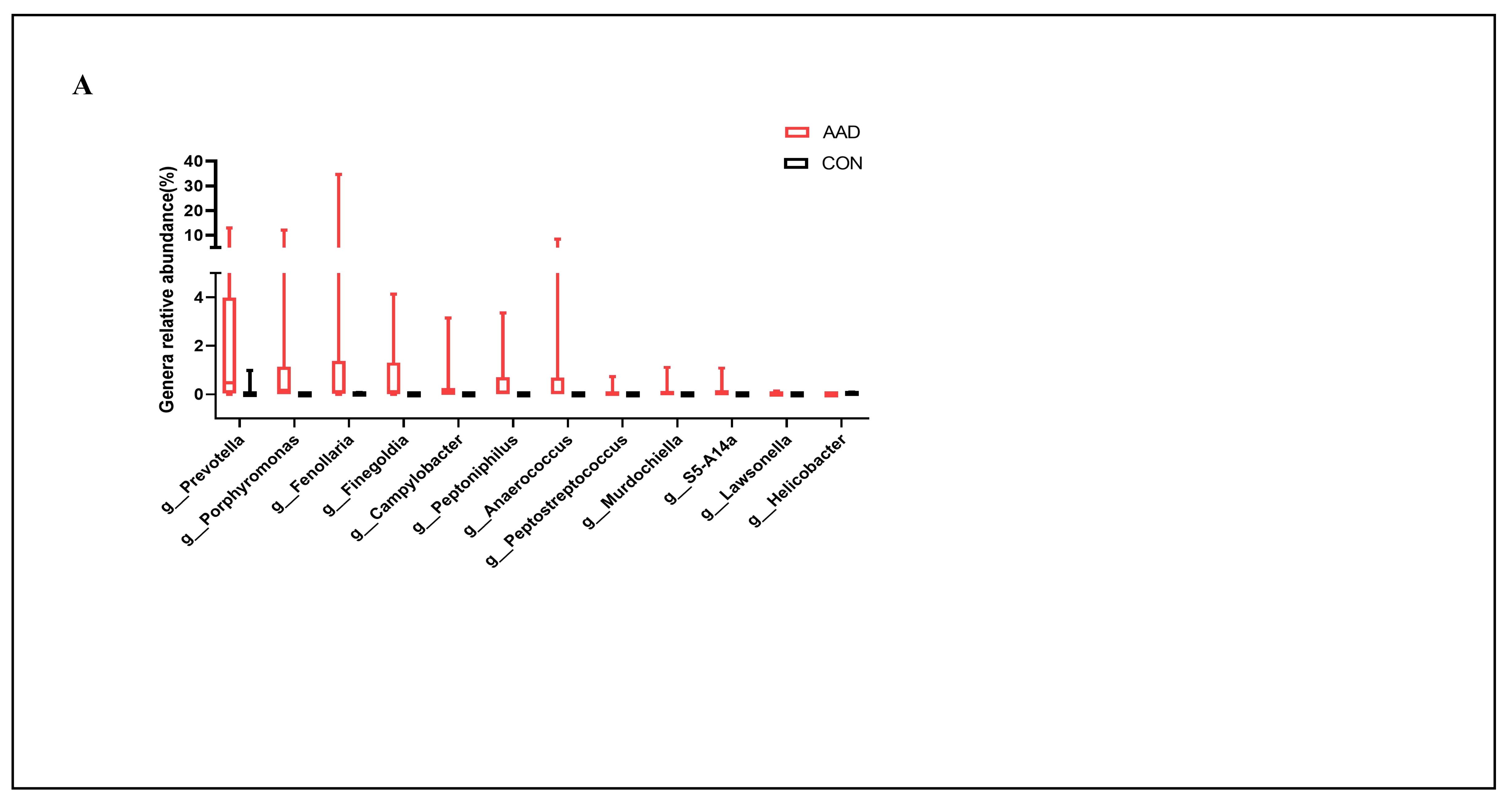

Supplement: Supplementary file 5 [file Image_2.jpg]
